# Prognostic Performance of Shock Index, Whole-Blood Lactate Concentration, Rectal–Interdigital Temperature Gradient, and ATT Score in Cats with Trauma-Induced Shock

**DOI:** 10.3390/ani16152410

**Published:** 2026-08-05

**Authors:** Sena Yazıcı Özcan, Kurtuluş Parlak

**Affiliations:** Department of Surgery, Faculty of Veterinary Medicine, Selcuk University, Selçuklu, 42130 Konya, Türkiye; senayazicivet@gmail.com

**Keywords:** feline trauma, shock index, whole-blood lactate, rectal–interdigital temperature gradient, Animal Trauma Triage score

## Abstract

Trauma-induced shock is a frequent and life-threatening complication in feline patients. Early detection of tissue hypoperfusion is fundamental for improving clinical outcomes; however, due to species-specific physiological responses in cats, conventional markers like the shock index often fail to accurately reflect shock severity. This prospective clinical study aimed to evaluate the prognostic value of the shock index for predicting mortality in traumatized cats and to analyze the relationship between clinical outcome and whole-blood lactate concentrations, the rectal–interdigital temperature gradient (RISG), and the Animal Trauma Triage (ATT) score. Fifty cats presenting with acute trauma were evaluated upon admission and at subsequent time points. Clinical shock was strictly defined by the presence of at least three markers of poor perfusion, including altered mentation, abnormal heart rate, poor peripheral pulses, and hypotension. Data were analyzed using receiver operating characteristic (ROC) curves and multivariable logistic regression. The Shock Index did not significantly distinguish survivors from nonsurvivors at any time point and showed no significant prognostic performance in ROC analysis (*p* > 0.05). Whole-blood lactate concentration showed modest prognostic discrimination (AUC = 0.678; 95% CI: 0.508–0.848; *p* = 0.041), with an optimal cut-off value of ≥4.46 mmol/L. However, the wide confidence interval and its lower limit close to 0.5 indicate considerable uncertainty, and this finding should be considered preliminary. The rectal–interdigital temperature gradient showed the highest prognostic performance (AUC = 0.804; 95% CI: 0.677–0.931; *p* < 0.001), with an optimal cut-off value of <2.05 °C. In multivariable logistic regression, both RISG (OR = 5.016; 95% CI: 1.783–14.109; *p* = 0.002) and whole-blood lactate concentration (OR = 0.567; 95% CI: 0.374–0.859; *p* = 0.007) were independently associated with in-hospital survival. These findings suggest that RISG may provide useful prognostic information, whereas whole-blood lactate concentration may offer complementary but preliminary prognostic information and should be interpreted cautiously. The Shock Index showed limited value for predicting in-hospital outcome in traumatized cats.

## Simple Summary

Shock following trauma in cats is a life-threatening condition in which inadequate tissue perfusion results in insufficient oxygen delivery to vital organs. Rapid identification of high-risk patients is essential for timely treatment. Although the Shock Index, calculated from heart rate and blood pressure, is widely used to assess circulatory compromise, our findings indicate that this index has limited prognostic value in traumatized cats because feline cardiovascular responses to shock differ from those of dogs. In contrast, RISG, a simple measure of the temperature difference between the body core and the paw, provided stronger prognostic information, whereas whole-blood lactate concentration showed modest prognostic discrimination with considerable uncertainty. A low RISG and a higher whole-blood lactate concentration were associated with an increased risk of an adverse in-hospital outcome; however, the prognostic finding for lactate should be considered preliminary. These findings suggest that assessing RISG alongside whole-blood lactate concentration may assist early risk stratification in traumatized cats, although further validation in larger cohorts is warranted.

## 1. Introduction

Shock is a systemic circulatory disorder characterized by an imbalance between oxygen delivery and cellular metabolic requirements, necessitating prompt clinical intervention [1]. In veterinary emergency medicine, the early detection of tissue hypoperfusion is of paramount importance for optimizing resuscitation strategies and improving clinical outcomes. Clinicians traditionally rely on vital parameters, such as heart rate (HR) and systolic blood pressure (SBP), to evaluate a patient’s hemodynamic status. Additionally, physical examination findings including mucous membrane color, capillary refill time, body temperature, and pulse quality are widely utilized as clinical indicators of circulatory failure [2].

However, the reliance on conventional vital parameters can be misleading, particularly in feline populations. Cats exhibit distinct physiological responses to systemic circulatory stress compared to dogs. For instance, rather than the compensatory tachycardia typically observed in dogs during shock, cats may exhibit bradycardia or normocardia due to the activation of the cardiac sympathovagal reflex [1]. Furthermore, the limited information provided by parameters such as mean arterial pressure (MAP) regarding true cellular oxygenation has prompted clinicians to seek more sensitive metabolic markers of tissue perfusion [2,3]. In this context, whole-blood lactate concentration is widely used as a metabolic marker of circulatory stress and impaired oxygen utilization; however, hyperlactatemia may develop through multiple mechanisms and is not specific to anaerobic metabolism or tissue hypoxia [2,4]. Recent preclinical trauma research has also demonstrated the feasibility of rapid serial whole-blood lactate assessment using point-of-care devices, supporting the practical application of lactate monitoring in acute trauma settings [5]. Nevertheless, because lactate concentration can be influenced by acute stress and struggling during restraint or blood collection, this parameter should be interpreted together with hemodynamic and clinical findings [1,6]. Moreover, lactate measurements may not always provide a definitive marker in every clinical scenario; studies on septic cats suggest that shock can manifest in different phenotypes—such as “dysoxic,” “cryptic,” or “vasoplegic”—depending on the presence of hypotension and hyperlactatemia [7].

To address these diagnostic challenges and detect occult hypoperfusion (occult shock), the Shock Index (SI) has been developed by calculating the ratio of heart rate to systolic blood pressure (SI = HR/SBP), effectively integrating cardiovascular compensatory mechanisms into a single index. Studies conducted on canine subjects have validated the utility of SI as an indicator of hemorrhagic shock, acute blood loss, and traumatic injury [8,9,10,11,12]. Despite this evidence-based utilization in dogs, research focusing on the clinical efficacy of SI in feline populations remains in its early stages. Fadel et al. [1] reported that SI was significantly elevated in cats presenting with shock compared to a control group, proposing a cut-off value of 1.54.

Despite the recent validation of shock index as a diagnostic marker of shock in cats [1], evidence regarding its prognostic significance remains lacking. In particular, no prospective studies have investigated whether SI can predict survival outcomes in feline trauma patients or whether it performs comparably to markers more directly associated with tissue perfusion, such as whole-blood lactate concentration or the rectal–interdigital temperature gradient (RISG). This knowledge gap is clinically relevant because early identification of high-risk trauma patients remains one of the major challenges in feline emergency and critical care medicine.

In addition to the Shock Index, composite trauma scoring systems have been developed to standardize injury severity assessment. The Animal Trauma Triage (ATT) score evaluates patients across six categories, with higher scores indicating more severe trauma and increased mortality risk [13]. Its prognostic performance has been validated in injured cats through a large multicenter Veterinary Committee on Trauma (VetCOT) registry study [14]. Similarly, a prospective study involving 25 cats with high-rise syndrome identified the ATT score as the only variable independently associated with mortality, further supporting its prognostic relevance in feline trauma patients [15]. More recently, a prospective study of dogs and cats with polytraumatic injuries demonstrated strong prognostic performance of the ATT score for predicting survival, further supporting its use in the early assessment of veterinary trauma patients [16]. A study evaluating 184 traumatized dogs and cats also reported an overall mortality rate of 35.3% and identified an ATT score ≥ 5 as a useful threshold for recognizing high-risk patients (OR = 4.207) [17]. Collectively, these findings support the use of ATT as a practical triage tool for early risk stratification, particularly when interpreted alongside physiological, laboratory, and clinical assessment findings.

Therefore, the primary aim of this prospective study was to evaluate the prognostic performance of the Shock Index (SI) in cats with trauma-induced shock and to examine the associations of whole-blood lactate concentration, the rectal–interdigital temperature gradient (RISG), and the Animal Trauma Triage (ATT) score with in-hospital outcome. We hypothesized that SI would show limited prognostic discrimination, whereas thermal, metabolic, and trauma-severity variables would be associated with clinical outcome. By examining these relationships, we sought to characterize the prognostic utility of these readily available parameters in feline trauma-induced shock. The findings of this study may contribute to improved early risk stratification and clinical decision-making in traumatized cats.

## 2. Materials and Methods

### 2.1. Materials

#### 2.1.1. Study Population and Inclusion/Exclusion Criteria

This prospective observational clinical study was conducted on 50 client-owned cats presented to the Emergency Service of the Department of Surgery, Faculty of Veterinary Medicine, Selçuk University, with acute traumatic injuries. To be included in the study, cats had to present within 24 h of the traumatic event and undergo an initial clinical assessment upon admission to the emergency service. The diagnosis of shock was based on the presence of at least three indicators of impaired tissue perfusion and circulatory compromise, including altered mentation (e.g., obtunded or stuporous), abnormal heart rate (tachycardia > 220 bpm or bradycardia < 140 bpm), pale or muddy mucous membranes, prolonged capillary refill time (>2 s), weak or absent peripheral pulses, hypothermia, and/or hypotension (systolic blood pressure < 90 mmHg) [2]. Fulfillment of at least three of these predefined criteria at admission was a mandatory inclusion requirement; therefore, all 50 enrolled cats had clinical evidence of shock at presentation. Hypotension represented one component of the composite clinical shock criteria and was not, by itself, a mandatory requirement for enrollment. Cats with a history of chronic kidney disease, hyperthyroidism, clinically relevant cardiac disease, neoplasia, or systemic inflammatory disorders were excluded. Furthermore, cats that had received medical treatment prior to referral, including intravenous fluid therapy, vasopressors, or non-steroidal anti-inflammatory drugs, were excluded to preserve baseline hemodynamic and metabolic measurements [18]. Written informed owner consent was obtained for all enrolled cats. The study was conducted in accordance with institutional animal welfare guidelines and was approved by the Ethics Committee of Selçuk University Faculty of Veterinary Medicine (protocol no. 2025/77; approved on 2 May 2025).

#### 2.1.2. Clinical and Hemodynamic Assessment

To determine the severity of trauma, the Animal Trauma Triage (ATT) scoring system and a comprehensive physical examination were performed for each patient immediately upon admission. All ATT assessments were performed by the same investigator. The ATT score evaluated patients across six categories: perfusion, cardiovascular, respiratory, ocular/musculoskeletal/integumentary, skeletal, and neurological parameters [13,14]. During respiratory rate assessment, stress- or pain-related rapid breathing/panting was not systematically distinguished from tachypnea associated with hypoxia, hypoperfusion, or traumatic respiratory injury, and panting was not recorded as a separate variable. Vital signs including heart rate, respiratory rate, systolic blood pressure, rectal temperature, interdigital temperature, and arterial oxygen saturation (SpO_2_) were recorded under conditions minimizing environmental stress, with the patient positioned in the most comfortable posture tolerated. The rectal–interdigital temperature gradient (RISG) was calculated as the difference between rectal and interdigital temperatures (RISG = T_rectal − T_interdigital), as originally described in dogs by Schaefer et al. [19] and subsequently applied in cats by Fadel et al. [1].

#### 2.1.3. Blood Pressure Measurement and Shock Index Calculation

Systolic blood pressure (SBP) was measured noninvasively using a Doppler ultrasonic flow detector (Parks Medical Electronics, Aloha, OR, USA). Cuff selection, measurement site, patient positioning, and efforts to minimize environmental stress followed the general technical recommendations of the American College of Veterinary Internal Medicine (ACVIM) consensus guidelines for blood pressure assessment [20,21]. An appropriately sized cuff, corresponding to approximately 40% of the circumference of the measurement site, was placed on the antebrachium. However, because of the emergency condition of the study population and the need for rapid serial assessment, the complete consensus protocol of discarding the first measurement and averaging multiple consecutive consistent readings could not be implemented. At each evaluation time point, Doppler measurements were attempted until a stable signal and a clinically acceptable value were obtained. If excessive patient movement or environmental stress interfered with the measurement, stressors were minimized and the measurement was repeated. Only the final accepted SBP value was recorded rather than the average of multiple measurements. This value was subsequently used to calculate the Shock Index (SI) as the ratio of heart rate to systolic blood pressure (SI = HR/SBP) [1]. To evaluate temporal changes in hemodynamic status, HR and SBP were recorded and SI was calculated at four predefined target time points relative to hospital admission: T1 (admission, 0 min), T2 (approximately 20 min after admission), T3 (approximately 40 min after admission), and T4 (approximately 60 min after admission). These time points represent elapsed time from admission rather than fixed intervals from the preceding measurement. Minor deviations of several minutes from the target times occurred because emergency diagnostic and therapeutic procedures took priority according to each patient’s clinical condition. Initial triage assessment and blood sampling were completed after T1, and emergency stabilization was subsequently initiated according to individual clinical requirements. Resuscitative and supportive interventions continued as clinically indicated between T2, T3, and T4.

#### 2.1.4. Laboratory Analyses

Immediately upon admission, 0.5–0.8 mL of venous blood was collected from the cephalic vein using a heparinized syringe and subsequently transferred into a K_3_EDTA tube. The sample was gently mixed and analyzed without centrifugation. Hematological parameters, including white blood cell count (WBC), red blood cell count (RBC), hemoglobin concentration (HGB), and hematocrit (HCT), were analyzed using an automated veterinary hematology analyzer (MS4e, Melet Schloesing Laboratoires, Osny, France). An aliquot of the same venous whole-blood sample was analyzed immediately using a blood gas analyzer (ABL90 FLEX, Radiometer Medical ApS, Brønshøj, Copenhagen, Denmark) to determine pH, base excess (BE), and whole-blood lactate concentration (LAC) [2]. Whole-blood lactate concentration was measured only at admission (T1) to characterize the initial metabolic status before substantial therapeutic intervention.

#### 2.1.5. Treatment Protocol and Patient Grouping

Following the initial clinical examination, triage assessment, and blood sampling, all cats were managed according to a standardized institutional trauma and shock resuscitation protocol, with treatment adjusted according to individual clinical requirements. In accordance with current recommendations for feline emergency and critical care [2,7], the protocol included isotonic crystalloid fluid therapy for volume resuscitation, analgesic treatment, primarily opioid-based when clinically indicated, and supplemental oxygen therapy to maintain adequate arterial oxygen saturation. Patients were hospitalized in the intensive care unit and monitored until clinical stabilization, surgical intervention when indicated, discharge, or death.

The primary clinical outcome was survival to hospital discharge. Cats that died during hospitalization, irrespective of the time elapsed after admission, were classified as nonsurvivors (*n* = 17), whereas cats discharged alive were classified as survivors (*n* = 33). These two outcome groups were used for all prognostic analyses.

### 2.2. Statistical Analysis

All statistical analyses were performed using IBM SPSS Statistics 27 (IBM Corp., Armonk, NY, USA). Graphical representations, including Shock Index distribution plots and receiver operating characteristic (ROC) curves, were generated using Python 3.12 with the Matplotlib version 3.10.8 library. Statistical significance was established at *p* < 0.05.

The distribution of continuous variables was assessed using the Shapiro–Wilk test. For the Shock Index, normality was assessed directly at each time point within each outcome group using the Shapiro–Wilk test. Variables fulfilling the assumption of normality were compared between the nonsurvivor and survivor group using the independent-samples *t*-test, with homogeneity of variance evaluated using Levene’s test. Variables that did not meet the assumption of normality were compared using the Mann–Whitney U test. The association between trauma mechanism (high-rise syndrome or vehicular trauma) and in hospital outcome was explored using Fisher’s exact test. No adjustment for multiple comparisons was applied to the univariate between-group comparisons presented in Table 1; therefore, the reported *p*-values are unadjusted.

Longitudinal changes within each group were evaluated using mixed repeated-measures analysis of variance (ANOVA) for normally distributed variables, with Greenhouse–Geisser correction applied when the assumption of sphericity was violated according to Mauchly’s test. The normality of the residuals from the repeated-measures model was also evaluated using the Shapiro–Wilk test. Non-normally distributed repeated measurements were analyzed using the Friedman test followed by Bonferroni-adjusted Wilcoxon signed-rank post hoc tests.

Associations between Shock Index and clinical or laboratory variables were evaluated using Spearman’s rank correlation coefficient. A sensitivity analysis was performed to estimate the minimum detectable absolute correlation coefficient, assuming a two-sided α of 0.05 and a statistical power of 80%. The prognostic performance of Shock Index, whole-blood lactate concentration, and the rectal–interdigital temperature gradient (RISG) was assessed using receiver operating characteristic (ROC) curve analysis, and optimal cut-off values were determined using the Youden index. Independent predictors of clinical outcome were identified using binary logistic regression. Based on the results of the univariable and ROC analyses, whole-blood lactate concentration and RISG were entered into the multivariable logistic regression model. Model calibration was assessed using the Hosmer–Lemeshow goodness-of-fit test, and explanatory performance was evaluated using the Nagelkerke R^2^. Model complexity was additionally evaluated using the events-per-variable (EPV) ratio, with 10 EPV considered a traditional reference threshold rather than an absolute criterion for logistic regression analysis [22,23].

## 3. Results

### 3.1. Patient Characteristics and Demographic Data

The study population comprised 50 client-owned cats, including 25 females (50.0%) and 25 males (50.0%), with a mean age of 16.7 ± 14.1 months (range: 3–60 months). Based on age, 24 (48.0%) cats were younger than 1 year, 17 (34.0%) were between 1 and 3 years of age, and 9 (18.0%) were older than 3 years.

Domestic Shorthair was the most frequently represented breed (*n* = 32, 64.0%), followed by British Shorthair (*n* = 8, 16.0%), Turkish Angora (*n* = 5, 10.0%), Bombay (*n* = 3, 6.0%), and Scottish Fold (*n* = 2, 4.0%). Among the Domestic Shorthair cats, four had a calico coat pattern, two had a tuxedo coat pattern, and one had a blue-point coat pattern. These coat patterns were recorded separately from breed classification.

The primary cause of trauma was high-rise syndrome (*n* = 34, 68.0%), followed by vehicular trauma (*n* = 16, 32.0%). In an exploratory subgroup comparison, in-hospital mortality was 26.5% (9/34) among cats with high-rise syndrome and 50.0% (8/16) among cats sustaining vehicular trauma. This difference was not statistically significant (Fisher’s exact test, *p* = 0.121). The mean interval between the traumatic event and admission to the emergency service was 5.7 ± 5.7 h (range: 0.5–21.0 h). Overall, 33 cats (66.0%) survived to hospital discharge, whereas 17 (34.0%) died during hospitalization.

### 3.2. Comparison of Single-Point Clinical and Laboratory Parameters

Baseline clinical and laboratory characteristics of survivor and nonsurvivor cats are presented in Table 1. Significant differences were observed between the two groups for the rectal–interdigital temperature gradient (RISG), pH, base excess, Animal Trauma Triage (ATT) score, rectal temperature, white blood cell (WBC) count, and whole-blood lactate concentration (all *p* < 0.05). Among these variables, RISG, pH, base excess, and ATT score demonstrated the strongest statistical differences (*p* < 0.001). In contrast, hematocrit (HCT), red blood cell (RBC) count, hemoglobin (HGB), and interdigital temperature did not differ significantly between survivors and nonsurvivors (*p* > 0.05).

### 3.3. Analysis of Hemodynamic and Respiratory Parameters

#### 3.3.1. Visual Distribution of the Shock Index

**Figure 1:**
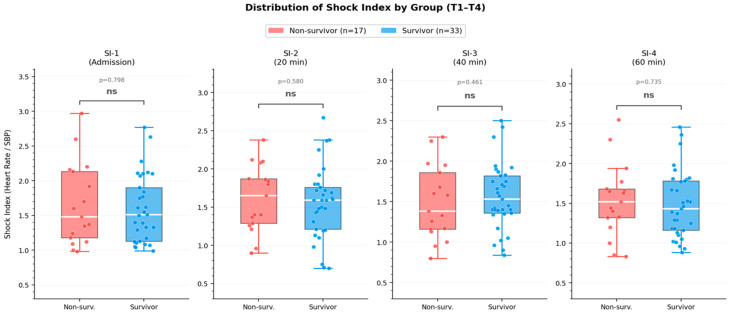
Distribution of Shock Index (SI) values in nonsurvivor and survivor cats at four measurement time points: T1 (admission, 0 min), T2 (approximately 20 min after admission), T3 (approximately 40 min after admission), and T4 (approximately 60 min after admission). Minor deviations of several minutes from the target times occurred because of patient-specific emergency procedures. Boxes represent the interquartile range (IQR), horizontal lines within the boxes indicate the medians, whiskers extend to the most extreme values within 1.5 × IQR, and dots represent individual observations. No statistically significant differences in SI were detected between the groups at any time point (all *p* > 0.05). The displayed *p*-values were obtained using two-sided Mann–Whitney U tests comparing nonsurvivors and survivors at each measurement time point. ns: not significant.

Figure 1 illustrates the distribution of Shock Index (SI) values at the four predefined measurement time points (T1–T4) in survivor and nonsurvivor cats. SI values showed substantial overlap between the two groups at all time points, with comparable median values and a wide dispersion of individual observations. No apparent separation between survivors and nonsurvivors was observed based on the visual distribution of SI.

#### 3.3.2. Comparison of Respiratory Rate and Oxygen Saturation

Time-dependent changes in respiratory rate (RR) and arterial oxygen saturation (SpO_2_) in survivor and nonsurvivor cats are summarized in Table 2. No significant differences were observed between the two groups in RR or SpO_2_ at admission (T1) (*p* > 0.05). At T2, the Nonsurvivor group exhibited a significantly lower respiratory rate than the Survivor group (*p* = 0.013). Similarly, arterial oxygen saturation was significantly lower in the Nonsurvivor group at T2 (*p* = 0.004) and T3 (*p* = 0.023). No significant differences were detected between the groups at the remaining time points (*p* > 0.05).

#### 3.3.3. Comparison of Heart Rate, Systolic Blood Pressure, and Shock Index

The comparison of heart rate (HR), systolic blood pressure (SBP), and Shock Index (SI) between survivor and nonsurvivor cats is presented in Table 3. At admission, 3 of the 50 cats (6.0%) had a systolic blood pressure below 90 mmHg, including 2 nonsurvivors (2/17; 11.8%) and 1 survivor (1/33; 3.0%). No statistically significant differences were observed between the two groups for HR, SBP, or SI at any of the four measurement time points (*p* > 0.05). Throughout the observation period, median SI values ranged from 1.38 to 1.65 across the outcome groups and measurement time points.

### 3.4. Longitudinal Analysis of Hemodynamic and Respiratory Parameters

#### 3.4.1. Heart Rate, Systolic Blood Pressure, and Shock Index: Repeated Measures ANOVA

The results of the repeated-measures ANOVA are summarized in Table 4, with descriptive statistics provided in Table 3. SI values were compatible with a normal distribution in all group-by-time combinations except T1 in the survivor group (W = 0.921, *p* = 0.019). Nevertheless, the residuals from the repeated-measures model did not significantly deviate from normality (W = 0.988, *p* = 0.080); therefore, the parametric repeated-measures ANOVA was retained. Because the assumption of sphericity was violated for heart rate (Mauchly’s test, *p* = 0.009), the Greenhouse–Geisser correction was applied. The assumption of sphericity was met for systolic blood pressure and Shock Index. No statistically significant effects were observed for time, group, or the group × time interaction for heart rate, systolic blood pressure, or Shock Index (all *p* > 0.05). Mean values for these variables remained relatively stable in both survivor and nonsurvivor groups throughout the four measurement time points.

#### 3.4.2. Respiratory Rate and Oxygen Saturation: Longitudinal Analysis

The results of the Friedman test and descriptive statistics are presented in Table 5. In the Survivor group, arterial oxygen saturation (SpO_2_) demonstrated a statistically significant increase from T1 to T4 (*p* = 0.010); however, post hoc analysis with Bonferroni correction revealed no significant differences between individual time-point pairs. The respiratory rate (RR) in the Survivor group showed a decreasing trend that approached statistical significance (*p* = 0.053). In contrast, no significant time-dependent changes were observed for either parameter in the Nonsurvivor group (*p* > 0.05).

### 3.5. Correlation Analysis Between Shock Index and Clinical Parameters

Spearman correlation analysis was used to evaluate associations between Shock Index values at T1–T4 and ATT score, rectal temperature, interdigital temperature, RISG, and whole-blood lactate concentration. No statistically significant correlations were identified at any time point (all *p* > 0.05; Table 6). For the sample size of 50 cats, assuming a two-sided α of 0.05 and a statistical power of 80%, the minimum detectable absolute correlation coefficient was approximately |r| = 0.38. Therefore, the absence of statistically significant correlations does not exclude weaker associations.

### 3.6. ROC Curve Analysis and Prognostic Cut-Off Points

**Figure 2:**
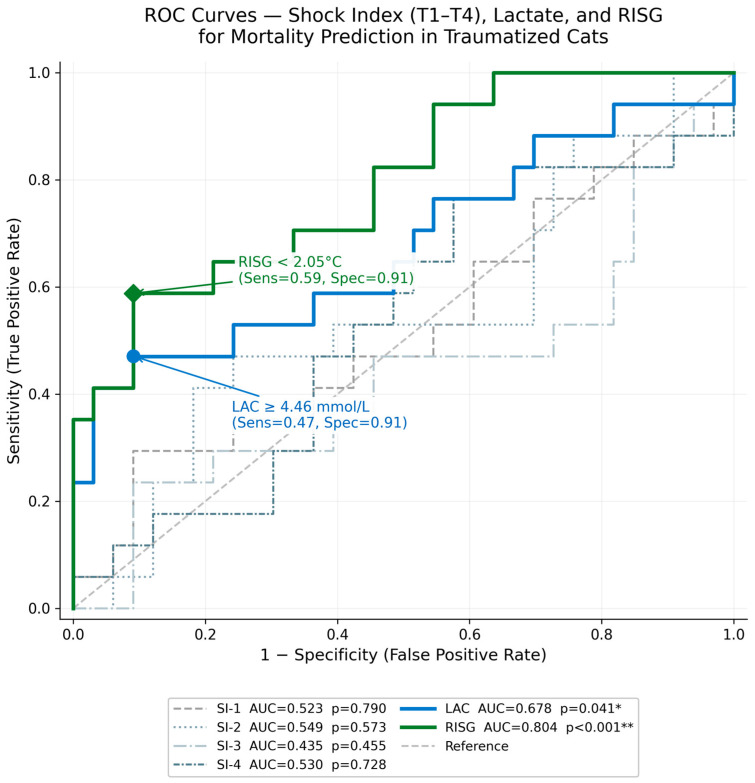
Receiver operating characteristic (ROC) curves for Shock Index (SI; T1–T4), whole-blood lactate concentration (LAC), and the rectal–interdigital temperature gradient (RISG) for predicting in-hospital mortality in traumatized cats. Circle: optimal cut-off value for LAC (≥4.46 mmol/L). Diamond: optimal cut-off value for RISG (<2.05 °C). AUC, area under the curve. * *p* < 0.05; ** *p* < 0.01.

The diagnostic performance of Shock Index (SI), whole-blood lactate concentration (LAC), and the rectal–interdigital temperature gradient (RISG) for predicting in-hospital mortality was evaluated using receiver operating characteristic (ROC) curve analysis. The results are presented in Table 7 and Figure 2. None of the SI measurements obtained at T1–T4 demonstrated statistically significant prognostic performance (all *p* > 0.05). The corresponding Youden index values ranged from 0.14 to 0.23. Whole-blood lactate concentration showed modest prognostic discrimination (AUC = 0.678; 95% CI: 0.508–0.848; *p* = 0.041), with an optimal cut-off value of ≥4.46 mmol/L (sensitivity, 47.1%; specificity, 90.9%). The wide confidence interval and its lower limit close to 0.5 indicate considerable uncertainty regarding the discriminatory ability of lactate despite the statistically significant AUC. RISG demonstrated significant prognostic performance (AUC = 0.804; 95% CI: 0.677–0.931; *p* < 0.001). The optimal cut-off value determined using the Youden index was <2.05 °C, corresponding to a sensitivity of 58.8% and a specificity of 90.9%.

### 3.7. Multivariable Logistic Regression Analysis

To identify independent predictors of clinical outcome, binary logistic regression analysis was performed. The dependent variable was defined as patient outcome, coded as nonsurvivor = 0 and Survivor = 1. Based on univariable analyses and ROC curve results, whole-blood lactate concentration (LAC) and the rectal–interdigital temperature gradient (RISG) were entered into the multivariable model. This two-variable model yielded an events-per-variable ratio of 8.5 (17 events/2 predictors). This EPV ratio is below the traditional rule-of-thumb of 10 and may increase the risk of model overfitting and instability of the coefficient estimates. Therefore, given the wide confidence intervals, the results of the multivariable model should be regarded as exploratory and interpreted cautiously.

The Omnibus test demonstrated that the model significantly improved prediction compared with the intercept-only model (χ^2^ = 24.961; df = 2; *p* < 0.001). The model explained 54.4% of the variance in clinical outcome (Nagelkerke R^2^ = 0.544). Model calibration was satisfactory according to the Hosmer–Lemeshow goodness-of-fit test (χ^2^ = 7.712; df = 8; *p* = 0.462). Overall classification accuracy was 82.0%, with a sensitivity of 70.6% for nonsurvivors and a specificity of 87.9% for survivors.

As shown in Table 8, both RISG and whole-blood lactate concentration were independently associated with clinical outcome. RISG was positively associated with survival (OR = 5.016; 95% CI: 1.783–14.109; *p* = 0.002), whereas whole-blood lactate concentration was negatively associated with survival (OR = 0.567; 95% CI: 0.374–0.859; *p* = 0.007).

## 4. Discussion

The primary objective of this prospective clinical study was to evaluate the prognostic performance of the Shock Index (SI) in cats with trauma-induced shock and to examine the associations of whole-blood lactate concentration, RISG, and the ATT score with in-hospital outcome. SI did not significantly distinguish survivors from nonsurvivors, whereas RISG and whole-blood lactate concentration were associated with clinical outcome. These findings demonstrate differences in the prognostic performance of the evaluated variables but should not be interpreted as evidence of physiological macro–microcirculatory decoupling. Neither RISG nor whole-blood lactate concentration provides a direct measurement of microcirculatory blood flow, and the absence of significant correlations with SI cannot establish dissociation between the macro- and microcirculations. Accordingly, the present findings are limited to the relative prognostic associations of these variables in traumatized cats. This cautious interpretation is consistent with critical-care literature indicating that systemic hemodynamic variables, microcirculatory assessment, peripheral perfusion findings, and blood lactate concentration provide complementary but non-interchangeable information about circulatory status [24,25,26].

Our baseline evaluations demonstrated that, although hematological variables (HCT, RBC, and HGB) did not differ significantly between survivors and nonsurvivors, several physiological and metabolic variables were markedly altered in the nonsurvivor group. In particular, pH and base excess exhibited the greatest degree of separation between groups (*p* < 0.001), suggesting more severe metabolic derangement in nonsurviving cats. This pattern is consistent with a retrospective emergency-room study of 185 cats, in which nonsurvivors had higher lactate concentrations, lower pH values, and more negative base deficits than survivors, while lactate concentration remained independently associated with mortality [27]. ATT scores were also significantly higher in nonsurvivors, consistent with recent prospective veterinary studies demonstrating the prognostic relevance of ATT for survival assessment in traumatized dogs and cats [15,16,17]. The rectal–interdigital temperature gradient (RISG) also differed significantly between groups, with survivors exhibiting higher RISG values than nonsurvivors. However, because a higher RISG represents a greater core-to-peripheral temperature difference, this finding should not be interpreted as evidence of better peripheral perfusion. Notably, survivors had a significantly higher rectal temperature, whereas interdigital temperature did not differ significantly between the groups. Therefore, the observed difference in RISG may have been influenced primarily by the lower core temperature of nonsurvivors rather than by greater peripheral cooling in survivors. Accordingly, RISG should be interpreted as an outcome-associated composite thermal measure rather than a direct indicator of preserved peripheral perfusion. Together, these findings indicate that metabolic disturbances and alterations in core-to-peripheral thermal patterns may provide clinically relevant prognostic information during the early assessment of feline trauma-induced shock [2].

When examining the longitudinal behaviour of cardiovascular variables, the visual and statistical distribution of the Shock Index across all four measurement time points (T1–T4) failed to demonstrate any prognostic capability in discriminating between survivors and non-survivors (*p* ≥ 0.05). The boxplot analysis (Figure 1) illustrated substantial overlap in the median and interquartile distributions of SI values between the two outcome groups at all four measurement time points. At first glance, these findings appear inconsistent with previous reports supporting the clinical utility of SI in veterinary emergency medicine. In particular, Fadel et al. [1] demonstrated that SI was significantly higher in cats presenting with shock than in healthy control cats and proposed a diagnostic cut-off value of 1.54 for identifying feline shock. However, the apparent discrepancy between their findings and ours is likely explained by fundamental differences in study objectives and patient populations. Whereas Fadel et al. [1] evaluated the diagnostic performance of SI for distinguishing shocked cats from non-shocked controls, the present study assessed the prognostic performance of SI exclusively within a population of traumatized cats already exhibiting clinical evidence of shock. Therefore, SI may retain value as a screening tool for the recognition of shock but lose discriminatory power when applied to outcome prediction among critically ill feline trauma patients. This interpretation is further supported by the fact that the shock population described by Fadel et al. [1] included heterogeneous disease processes such as urethral obstruction, metabolic disorders, neoplastic diseases, and trauma, whereas our cohort consisted exclusively of trauma patients. Consequently, our study evaluated SI under a more clinically homogeneous yet prognostically demanding scenario. In contrast to the diagnostic utility reported by Fadel et al. [1], SI failed to differentiate survivors from nonsurvivors in the present investigation. Similar limitations have also been observed in feline patients displaying atypical cardiovascular responses to shock. Furthermore, several studies in dogs have reported an association between elevated SI and both shock severity and mortality, particularly in patients with hemorrhagic injury, vehicular trauma, and head trauma [8,11,12]. Human trauma studies further illustrate the context-dependent prognostic performance of SI. SI was associated with mortality in patients undergoing emergency surgery for trauma, whereas a composite approach combining reverse SI with a simplified motor score provided stronger prognostic discrimination than SI alone in a large adult trauma cohort [28,29]. The limited prognostic performance of SI in our cohort may be attributed to the unique, often paradoxical feline counter-regulatory response to shock. Unlike dogs, which predictably develop tachycardia to sustain cardiac output during hypovolaemia, cats frequently manifest normocardia or bradycardia as a consequence of enhanced vagal stimulation and species-specific cardiovascular responses to shock [1]. Because the Shock Index (SI) is calculated as the ratio of heart rate to systolic blood pressure, these atypical heart rate responses may mathematically attenuate changes in SI, thereby reducing its ability to accurately reflect the severity of circulatory compromise in traumatized feline patients. In addition, only 3 of the 50 cats met the hypotension criterion at admission, and the mean SBP values in both outcome groups remained well above the 90 mmHg threshold. Because SBP constitutes the denominator of the Shock Index, the predominance of preserved and similar SBP values between the groups may have contributed to the limited prognostic discriminatory ability of SI. In addition, the accuracy of SI depends directly on the reliability of the SBP value used as its denominator. Because only one accepted Doppler-derived SBP measurement was retained at each time point rather than the average of several consistent readings, measurement variability may have been propagated into the calculated SI values. This concern is supported by a prospective study in anesthetized cats that demonstrated poor agreement and wide limits of agreement between Doppler-derived and directly measured arterial blood pressure values [30]. This methodological factor may have contributed to the substantial overlap between groups and the limited prognostic discrimination of SI. Therefore, the observed performance of SI may reflect both biological characteristics of feline trauma-induced shock and limitations of the blood pressure measurement protocol. In the present study, heart rate, systolic blood pressure, and SI did not significantly differ between survivors and nonsurvivors, whereas RISG and whole-blood lactate concentration were associated with clinical outcome. This discrepancy indicates that these variables provided different levels of prognostic information within the present cohort. However, it does not establish physiological dissociation between macro- and microcirculatory function because microcirculatory blood flow was not directly measured. Repeated-measures analysis similarly demonstrated no significant effects of time, group, or the group × time interaction for heart rate, systolic blood pressure, or SI during the initial observation period. Collectively, these findings suggest that apparently stable conventional hemodynamic variables may have limited value for outcome prediction in traumatized cats and should therefore be interpreted together with the broader clinical and metabolic assessment. Although apparently stable heart rate or blood pressure should not be considered sufficient to exclude ongoing hypoperfusion in shock states [7], the present study did not directly measure microcirculatory blood flow. Therefore, the observed differences in prognostic performance cannot be interpreted as evidence of physiological macro–microcirculatory decoupling. Conversely, the intra-group longitudinal analysis of respiratory variables using the Friedman test (Table 5) provided additional insight into the clinical course of the patients. In the survivor group, arterial oxygen saturation (SpO_2_) showed a significant overall change over time (*p* = 0.010), although no individual pairwise comparison remained significant after Bonferroni correction. Respiratory rate did not change significantly over time (*p* = 0.053). In contrast, neither respiratory rate nor SpO_2_ changed significantly over time in the nonsurvivor group (*p* > 0.05). These findings suggest that persistent impairment of oxygenation may be associated with an unfavourable clinical course despite emergency stabilization and supportive treatment.

Spearman rank correlation analysis (Table 6) showed no statistically significant correlations between SI at any time point and lactate, rectal temperature, or RISG (*p* > 0.05). These findings indicate that SI did not closely track the evaluated thermal and metabolic variables in this cohort. However, the absence of significant correlations should not be interpreted as evidence of physiological macro–microcirculatory decoupling or as proof that SI cannot reflect microcirculatory blood flow or tissue oxygenation. Given that the minimum detectable absolute correlation coefficient was approximately |r| = 0.38, weaker associations between SI and the evaluated clinical variables cannot be excluded and should be investigated in larger cohorts. Direct measurements of microcirculatory perfusion would be required to determine the relationship between SI and microcirculatory function.

Our findings are in agreement with those of Zollo et al. [18], who reported that admission shock index did not discriminate survivors from nonsurvivors in dogs diagnosed with shock. In that study, serial lactate-derived variables demonstrated greater prognostic utility than a single admission SI measurement. Although our study focused on trauma-induced shock in cats, both investigations suggest that static macrohemodynamic indices may have limited value for outcome prediction when compared with variables more closely related to tissue perfusion and metabolic status.

In terms of prognostic discrimination, receiver operating characteristic (ROC) analysis demonstrated that the rectal–interdigital temperature gradient (RISG) exhibited the strongest predictive performance among the variables included in the ROC analysis (AUC = 0.804; *p* < 0.001). The optimal cut-off value of RISG < 2.05 °C yielded a sensitivity of 58.8% and a specificity of 90.9%, indicating good discriminatory ability for identifying cats at increased risk of in-hospital mortality. Whole-blood lactate concentration showed modest prognostic discrimination (AUC = 0.678; 95% CI: 0.508–0.848; *p* = 0.041), with an optimal cut-off value of ≥4.46 mmol/L. However, because the confidence interval was wide and its lower limit was close to 0.5, this finding should be considered preliminary and interpreted cautiously. Although lactate showed high specificity (90.9%), its modest sensitivity (47.1%) indicates that an increased lactate concentration may indicate a higher risk of an adverse outcome when present, whereas a normal lactate concentration cannot reliably exclude an adverse clinical outcome. The context-dependent prognostic value of lactate is also supported by a prospective study of 123 hospitalized cats, in which neither admission lactate concentration nor serial lactate measurements were significantly associated with survival to discharge [31]. These findings are consistent with previous studies demonstrating that whole-blood lactate concentration reflects tissue hypoperfusion and provides valuable prognostic information but should be interpreted in conjunction with clinical indicators of tissue perfusion rather than as an isolated marker [2,18]. The concordance between the ROC analysis and the multivariable logistic regression model suggests that whole-blood lactate concentration may provide complementary prognostic information; however, this finding requires validation in larger cohorts.

Multivariable logistic regression showed that both the rectal–interdigital temperature gradient (RISG) and whole-blood lactate concentration were independently associated with clinical outcome. Higher RISG was associated with greater odds of survival, whereas higher whole-blood lactate concentration was associated with lower odds of survival. However, because RISG is a composite thermal measure influenced by both rectal and interdigital temperatures, its association with outcome should not be interpreted as direct evidence of peripheral perfusion. These variables may provide complementary prognostic information, but their combined clinical utility requires validation in larger cohorts.

Schaefer et al. [19] originally evaluated the rectal–interdigital temperature gradient as a diagnostic marker of circulatory shock in dogs and reported that it was increased in dogs with shock and positively correlated with SI (ρ = 0.617; *p* < 0.001), consistent with the conventional interpretation that a greater core-to-peripheral temperature gradient reflects peripheral vasoconstriction. In cats, however, Fadel et al. [1] reported that the rectal interdigital temperature gradient did not significantly differ between healthy cats and cats classified as being in shock, although individual rectal and peripheral temperatures differed between groups. In the present study, lower RISG was associated with nonsurvival, and RISG remained independently associated with clinical outcome together with whole-blood lactate concentration in the multivariable analysis. This direction of association was counterintuitive from the perspective of peripheral vasoconstriction. Because nonsurvivors had a significantly lower rectal temperature, whereas interdigital temperature did not differ significantly between the outcome groups, the lower RISG observed in nonsurvivors may primarily reflect lower core temperature or altered thermoregulatory status rather than better peripheral perfusion in survivors. Differences in trauma severity, stage of shock, and time from injury to admission may also have contributed to this finding. Therefore, the present results support the potential prognostic value of RISG as a composite thermal measure but do not establish it as a direct indicator of peripheral perfusion or preserved microcirculatory function. Consistent with the observations of Reineke et al. [2], RISG should be interpreted together with metabolic and clinical variables rather than as an isolated measure of tissue perfusion. Serial evaluation of both rectal and interdigital temperatures in larger cohorts is required to clarify the physiological basis and prognostic direction of this association.

### Limitations of the Study

Several limitations should be considered when interpreting the results of this study. First, this prospective study included a relatively small cohort of 50 cats from a single veterinary teaching hospital, which may have limited the statistical power to detect independent associations for variables such as the Animal Trauma Triage (ATT) score and whole-blood lactate concentration in the multivariable logistic regression model. Because multiple univariate comparisons were performed in Table 1 without adjustment for multiple testing, an increased risk of Type I error cannot be excluded [32]. Therefore, the findings concerning WBC and whole-blood lactate concentration should be interpreted cautiously.

Second, systolic blood pressure was measured noninvasively using a Doppler ultrasonic flow detector, and only one clinically acceptable SBP value was recorded at each time point. This approach did not fully conform to consensus protocols recommending that the first reading be discarded and that several consecutive consistent measurements be averaged. Although patient movement and environmental stress were minimized and measurements were repeated when necessary until a stable Doppler signal was obtained, retaining a single selected value may have introduced potentially substantial random or selection-related measurement error. Because SBP constitutes the denominator of SI, any error in SBP measurement may have been propagated and amplified in the calculated SI value. This variability may have attenuated or exaggerated between-group differences and temporal changes and may therefore have contributed to the limited prognostic performance of SI observed in this study. Furthermore, peripheral vasoconstriction in severely hypotensive or hypothermic cats may have reduced the accuracy of Doppler-derived SBP measurements. Accordingly, the SI findings should be interpreted cautiously, and future studies should use standardized repeated-measurement protocols or invasive arterial blood pressure monitoring when feasible.

Third, stress- or pain-related rapid breathing could not be systematically distinguished from tachypnea associated with hypoxia, hypoperfusion, or traumatic respiratory injury. Therefore, the wide variability in respiratory rate, particularly at T1 in the survivor group, should be interpreted cautiously.

Fourth, the study population consisted exclusively of cats with trauma-induced shock, predominantly resulting from high-rise syndrome (68%) and vehicular trauma (32%). High-rise syndrome and vehicular trauma may be associated with different injury patterns and physiological consequences. Feline high-rise syndrome itself may result in a heterogeneous spectrum of injuries, including extremity fractures, pneumothorax, pulmonary contusions, and other forms of thoracic trauma [33]. Although the exploratory subgroup comparison did not demonstrate a statistically significant association between trauma mechanism and in-hospital outcome, mortality was numerically higher following vehicular trauma, and the small and unequal subgroup sizes limited statistical power. Therefore, residual confounding by trauma mechanism cannot be excluded, and mechanism-specific associations should be evaluated in larger cohorts. Furthermore, the observed relationships among Shock Index (SI), whole-blood lactate concentration, and the rectal–interdigital temperature gradient (RISG) may not be directly applicable to other forms of feline shock, including septic, cardiogenic, or primary hemorrhagic shock.

Fifth, the interval between the traumatic event and admission varied considerably (0.5–21.0 h). Because whole-blood lactate concentration and peripheral temperature gradients may change during the evolution of shock, this variability may have influenced the admission LAC and RISG values and introduced residual confounding. Time to admission was not included as a covariate in the multivariable model because the limited number of mortality events and the resulting risk of model overfitting precluded the reliable estimation of an additional predictor. Therefore, its potential influence cannot be excluded, and future studies should standardize the assessment time or account for this interval in analyses involving larger cohorts.

Sixth, RISG is a composite variable influenced by both rectal and interdigital temperatures and cannot independently distinguish peripheral vasoconstriction from changes in core temperature. In the present study, nonsurvivors had a lower rectal temperature, whereas interdigital temperature did not differ significantly between the outcome groups. Therefore, the association between lower RISG and nonsurvival may have been driven partly by core hypothermia or altered thermoregulation rather than by differences in peripheral perfusion. Furthermore, neither RISG nor whole-blood lactate concentration represents a direct measurement of microcirculatory blood flow, and no direct microcirculatory monitoring technique was used. Consequently, the differences in prognostic performance and the absence of significant correlations with SI cannot establish physiological macro–microcirculatory decoupling. The present findings should therefore be interpreted as prognostic associations rather than evidence of a specific circulatory mechanism. Serial assessment of both temperature components together with direct measures of microcirculatory perfusion is required in future studies.

Finally, whole-blood lactate concentration was measured only once at admission (T1). Serial lactate measurements and lactate clearance have been shown to provide additional prognostic information in critically ill veterinary patients, with a large emergency-room study reporting an association between hyperlactatemia and mortality in both dogs and cats [18,34]. Evidence from human critical care similarly indicates that peak lactate within the first 24 h may provide prognostic information in selected patient populations; in septic shock patients receiving continuous renal replacement therapy, peak lactate was independently associated with 28-day mortality [35]. Therefore, future prospective studies incorporating serial lactate assessment together with dynamic tissue perfusion markers may provide a more comprehensive evaluation of shock progression and clinical outcome in traumatized cats. In addition, clinical outcome in the present study was limited to survival to hospital discharge. Future studies evaluating long-term survival, post-discharge outcomes, and functional recovery would further clarify the prognostic utility of the evaluated parameters.

## 5. Conclusions

In conclusion, this study demonstrated differences in the prognostic performance of the evaluated clinical, thermal, and metabolic variables in cats with trauma-induced shock. Conventional hemodynamic parameters, particularly SI, did not distinguish survivors from nonsurvivors and showed no significant prognostic performance in ROC analysis. In contrast, RISG was associated with clinical outcome, whereas whole-blood lactate concentration showed more modest and uncertain prognostic discrimination. However, because neither RISG nor whole-blood lactate concentration directly measures microcirculatory blood flow, these findings should not be interpreted as evidence of macro–microcirculatory dissociation. Rather, they indicate that RISG and whole-blood lactate concentration may provide prognostic information that is not captured by SI in this patient population.

## Figures and Tables

**Table 1:** Comparison of baseline clinical and laboratory characteristics between nonsurvivor and Survivor groups.

Variable	Nonsurvivor Group (*n* = 17)	Survivor Group (*n* = 33)	*p*-Value
**RISG (°C)**	**2.10 ± 0.98**	**3.29 ± 0.93**	**<0.001**
HCT (%)	29.96 ± 6.32	32.78 ± 6.44	0.147
**pH**	**7.237 ± 0.102**	**7.371 ± 0.052**	**<0.001**
**Base Excess (mEq/L)**	**−12.47 ± 4.13**	**−8.10 ± 3.62**	**<0.001**
**Rectal Temp (°C)**	**38.10 (37.20–38.90)**	**38.70 (37.90–39.20)**	**0.002**
Interdigital Temp (°C)	35.50 (34.00–35.80)	35.60 (35.00–36.10)	0.636
**ATT Score**	**9.00 (6.00–12.00)**	**5.00 (4.00–7.00)**	**<0.001**
**WBC (×10^3^/µL)**	**12.61 (10.73–21.43)**	**10.53 (8.25–15.14)**	**0.008**
RBC (×10^6^/µL)	7.82 (6.67–9.89)	8.15 (7.05–9.87)	0.197
HGB (g/dL)	11.80 (9.50–14.50)	12.60 (10.70–14.30)	0.296
**LAC (mmol/L)**	**5.31 (2.08–7.66)**	**1.98 (1.48–2.94)**	**0.041**

Data are expressed as mean ± standard deviation (SD) for normally distributed variables (RISG, HCT, pH, base excess) or median (interquartile range; Q1–Q3) for non-normally distributed variables (rectal temperature, interdigital temperature, ATT score, WBC, RBC, HGB, LAC). Statistically significant values (*p* < 0.05) are indicated in bold. The *p*-values presented in Table 1 were not adjusted for multiple comparisons. Abbreviations: RISG, rectal–interdigital temperature gradient; HCT, hematocrit; WBC, white blood cell count; RBC, red blood cell count; HGB, hemoglobin; LAC, lactate; ATT, Animal Trauma Triage.

**Table 2:** Comparison of respiratory rate and oxygen saturation between Nonsurvivor and Survivor groups across four time points (Mann–Whitney U test).

Parameter	Time	Nonsurvivor Group (*n* = 17) Median (Q1–Q3)	Survivor Group (*n* = 33) Median (Q1–Q3)	*p*-Value	
Respiratory rate (breaths/min)	T1	37.0 (32.0–88.0)	72.0 (37.0–101.5)	0.179	**–**
	T2	35.0 (32.5–54.0)	56.0 (36.0–78.0)	0.013 *	*
	T3	41.0 (30.0–56.0)	45.0 (35.0–60.0)	0.505	**–**
	T4	42.0 (35.5–60.5)	44.0 (36.0–62.0)	0.894	**–**
Oxygen saturation (%)	T1	86.0 (63.5–91.0)	87.0 (83.5–93.5)	0.226	**–**
	T2	80.0 (64.5–91.0)	91.0 (86.0–94.0)	0.004 *	*
	T3	82.0 (73.0–93.5)	92.0 (88.5–96.0)	0.023 *	*
	T4	89.0 (76.5–96.0)	94.0 (88.5–97.0)	0.139	**–**

Q1: first quartile; Q3: third quartile. * *p* < 0.05. Statistically significant values are indicated in bold.

**Table 3:** Comparison of heart rate, systolic blood pressure, and Shock Index between nonsurvivor and survivor groups across four measurement time points.

Parameter	Time	Nonsurvivor Group (*n* = 17)	Survivor Group (*n* = 33)	*p*-Value
**Heart rate (beats/min)**	T1	203.3 ± 45.7	199.0 ± 39.1	0.730
	T2	201.8 ± 41.4	191.3 ± 36.5	0.361
	T3	196.1 ± 54.7	190.6 ± 40.1	0.685
	T4	195.8 ± 42.7	192.3 ± 36.1	0.764
**Systolic blood pressure (mmHg)**	T1	130.3 ± 30.9	130.5 ± 28.4	0.980
	T2	128.1 ± 21.8	129.2 ± 30.6	0.896
	T3	132.4 ± 17.0	124.8 ± 25.0	0.268
	T4	131.6 ± 24.5	134.7 ± 32.2	0.731
**Shock index**	T1	1.48 (1.18–2.13)	1.51 (1.13–1.90)	0.798
	T2	1.65 (1.29–1.87)	1.59 (1.21–1.76)	0.580
	T3	1.38 (1.16–1.86)	1.53 (1.36–1.82)	0.461
	T4	1.52 (1.32–1.68)	1.43 (1.16–1.78)	0.735

Heart rate and systolic blood pressure data are presented as mean ± standard deviation (SD), whereas Shock Index data are presented as median (interquartile range; Q1–Q3). Heart rate and systolic blood pressure were compared using independent-samples *t*-tests, and Shock Index was compared using two-sided Mann–Whitney U tests. No statistically significant differences were detected between the groups (all *p* > 0.05).

**Table 4:** Results of the repeated-measures ANOVA for heart rate, systolic blood pressure, and Shock Index.

Parameter	Mauchly’s *p*	Time Effect *p*	Group × Time *p*	Group Effect *p*
Heart rate (beats/min)	0.009 (GG)	0.398	0.863	0.575
Systolic blood pressure (mmHg)	0.074 (SA)	0.784	0.744	0.882
Shock index	0.703 (SA)	0.395	0.748	0.800

GG: Greenhouse–Geisser correction applied; SA: Sphericity assumed. Time effect: overall change from T1 to T4; Group × Time: interaction effect; Group effect: overall mean difference between groups.

**Table 5:** Longitudinal changes in respiratory rate and oxygen saturation within the nonsurvivor and survivor groups across four time points (Friedman test).

Parameter	Group	T1 Median (Q1–Q3)	T2 Median (Q1–Q3)	T3 Median (Q1–Q3)	T4 Median (Q1–Q3)	Friedman *p*
Respiratory rate (breaths/min) Survivor	Nonsurvivor	37.0 (32.0–88.0)	35.0 (32.5–54.0)	41.0 (30.0–56.0)	42.0 (35.5–60.5)	0.120
	72.0 (37.0–101.5)	56.0 (36.0–78.0)	45.0 (35.0–60.0)	44.0 (36.0–62.0)	0.053	
Oxygen saturation (%) Survivor	Nonsurvivor	86.0 (63.5–91.0)	80.0 (64.5–91.0)	82.0 (73.0–93.5)	89.0 (76.5–96.0)	0.064
	87.0 (83.5–93.5)	91.0 (86.0–94.0)	92.0 (88.5–96.0)	94.0 (88.5–97.0)	0.010 *	

Q1: first quartile; Q3: third quartile. * *p* < 0.05. Post hoc analysis with Bonferroni-corrected Wilcoxon test: Survivor group SpO_2_ T1–T4 raw *p* = 0.020; Bonferroni-corrected *p* = 0.120 (not significant).

**Table 6:** Spearman correlation coefficients between the shock index (T1–T4) and clinical/laboratory parameters.

Shock Index	Parameter	Spearman’s r	*p*-Value
**SI-1**	ATT score	0.194	0.177
	Rectal temperature	0.113	0.434
	Interdigital temperature	0.138	0.341
	RISG	0.059	0.685
	LAC	−0.057	0.692
**SI-2**	ATT score	0.220	0.125
	Rectal temperature	−0.093	0.520
	Interdigital temperature	−0.123	0.397
	RISG	0.001	0.993
	LAC	0.054	0.712
**SI-3**	ATT score	0.200	0.163
	Rectal temperature	0.031	0.832
	Interdigital temperature	0.039	0.786
	RISG	0.120	0.405
	LAC	−0.059	0.684
**SI-4**	ATT score	0.174	0.226
	Rectal temperature	−0.205	0.153
	Interdigital temperature	−0.262	0.067
	RISG	0.063	0.666
	LAC	0.185	0.199

ATT: Animal Trauma Triage score; RISG: rectal–interdigital temperature gradient; LAC: lactate. All *p*-values were >0.05.

**Table 7:** Receiver operating characteristic (ROC) curve analysis results for Shock Index (T1–T4), whole-blood lactate concentration, and the rectal–interdigital temperature gradient.

Variable	AUC	95% CI	*p*-Value	Cut-Off	Sensitivity	Specificity
Shock Index T1	0.523	0.345–0.702	0.790	2.125	0.294	0.909
Shock Index T2	0.549	0.373–0.725	0.573	1.820	0.412	0.818
Shock Index T3	0.435	0.253–0.617	0.455	1.945	0.235	0.909
Shock Index T4	0.530	0.358–0.703	0.728	1.305	0.765	0.424
Lactate (LAC)	0.678	0.508–0.848	0.041 *	4.460	0.471	0.909
RISG	0.804	0.677–0.931	<0.001 **	2.050	0.588	0.909

AUC: Area Under the Curve; CI: Confidence Interval. The cut-off point was determined using the Youden Index (Sensitivity + Specificity − 1). * *p* < 0.05, ** *p* < 0.01. Statistically significant *p*-values are indicated in bold.

**Table 8:** Multivariable logistic regression analysis identifying independent predictors of in-hospital survival in traumatized cats.

Variable	B	SE	Wald	*p*-Value	Odds Ratio (OR)	95% CI
LAC (mmol/L)	−0.568	0.212	7.182	**0.007**	0.567	0.374–0.859
RISG	1.613	0.528	9.342	**0.002**	5.016	1.783–14.109

Abbreviations: B, regression coefficient; SE, standard error; OR, odds ratio; CI, confidence interval; LAC, whole-blood lactate concentration; RISG, rectal–interdigital temperature gradient. The dependent variable was coded as nonsurvivor = 0 and survive = 1. Therefore, OR values > 1 indicate increased odds of survival, whereas OR values < 1 indicate decreased odds of survival (i.e., increased odds of in-hospital mortality). Model performance: Omnibus test: χ^2^ = 24.961, *p* < 0.001; Hosmer–Lemeshow test: χ^2^ = 7.712, *p* = 0.462; Nagelkerke R^2^ = 0.544. The model had an EPV ratio of 8.5 based on 17 outcome events and two predictors. Because this value is below the traditional rule-of-thumb of 10 EPV, the possibility of model overfitting and imprecise odds-ratio estimates cannot be excluded. Statistically significant *p*-values are indicated in bold.

## Data Availability

The data supporting the findings of this study are available from the corresponding author upon reasonable request. The data are not publicly available due to institutional and privacy considerations.

## Abbreviations

ATT	Animal Trauma Triage
AUC	Area Under the Curve
BE	Base Excess
CI	Confidence Interval
EPV	Events per Variable
HCT	Hematocrit
HGB	Hemoglobin
IQR	Interquartile Range
LAC	Whole-Blood Lactate Concentration
MAP	Mean Arterial Pressure
OR	Odds Ratio
RBC	Red Blood Cell Count
RISG	Rectal–Interdigital Temperature Gradient
ROC	Receiver Operating Characteristic
RR	Respiratory Rate
SBP	Systolic Blood Pressure
SD	Standard Deviation
SI	Shock Index
SPSS	Statistical Package for the Social Sciences
WBC	White Blood Cell Count
